# Recent advances in managing and understanding seborrheic keratosis

**DOI:** 10.12688/f1000research.18983.1

**Published:** 2019-08-28

**Authors:** Uwe Wollina

**Affiliations:** 1 Department of Dermatology and Allergology, Städtisches Klinikum Dresden, Dresden, 01067, Germany

**Keywords:** Seborrheic keratosis, diagnosis, pathogenesis, treatment

## Abstract

Seborrheic keratosis (SK) is a common benign epidermal tumor with predominance in adult patients. Whereas common SKs are more frequent in Caucasians, dermatosis papulosa nigra is more prevalent in patients with a Fitzpatrick skin type of at least 3. There seems to be a link between extrinsic skin aging and the occurrence of SK. Mutations of fibroblast growth factor receptor 3 and other signaling molecules are a frequent finding in SK lesions. However, this does not translate into any malignant potential. Viral infections are particularly common in genital lesions, although their pathogenetic relevance for SK is questionable. Different histologic and clinical subtypes have been identified. The great variability of SKs raises some difficulties in diagnosis. Dermoscopy is the preferred non-invasive diagnostic method, in particular to differentiate pigmented SKs from other pigment tumors, including cutaneous melanoma. Eruptive SKs can be a paraneoplastic condition known as the Leser–Trélat sign. New targeted cancer treatments can cause a pseudo-Leser–Trélat sign. The treatment in practice is mainly minor surgery, including cryosurgery, shave excisions, and laser-assisted removal. The medical approaches have only limited effects. Recently, two formulations for topical therapy have been evaluated: a product with 40% hydrogen peroxide (HP40) and an aqueous nitric–zinc complex. Based on clinical trials, HP40 seems to be a promising alternative to surgery, in particular for facial lesions.

## Introduction

Seborrheic keratoses (SKs) are very common benign epithelial skin tumors encountered in the adult population and show an increasing incidence with age, which reaches a peak at 60 years. The predilection zones of SKs are trunk and forehead. On the trunk, they follow the Langer’s lines ^1^. Rarely, SKs are seen in the external ear canal ^2^. SKs in the genital area may be misinterpreted as human papillomavirus (HPV) lesions or extramammary Paget’s disease ^3, 4^. Pendulating lesions may occur but they are uncommon ^5^. An important clinical sign is the formation of multiple horn pearls, which is neither seen in all types of SKs nor exclusive to SK ^1^.

## Non-invasive diagnosis beyond the naked eye

A definitive diagnosis of SK can be confusing with the naked eye because of the variability in clinical appearance of this condition. However, most cases of SK exhibit the typical dermoscopic findings of fissures and ridges, hairpin vessels with white halo, comedo-like openings, and milia-like cysts. The histopathologic counterparts of the dermoscopic findings are papillomatous epidermis, enlarged dermal capillaries, pseudo-horn cysts, and intraepidermal cysts ^6, 7^. Dermoscopy is of practical value in differentiating SKs from malignant melanoma ^8^.

**Figure 1.**
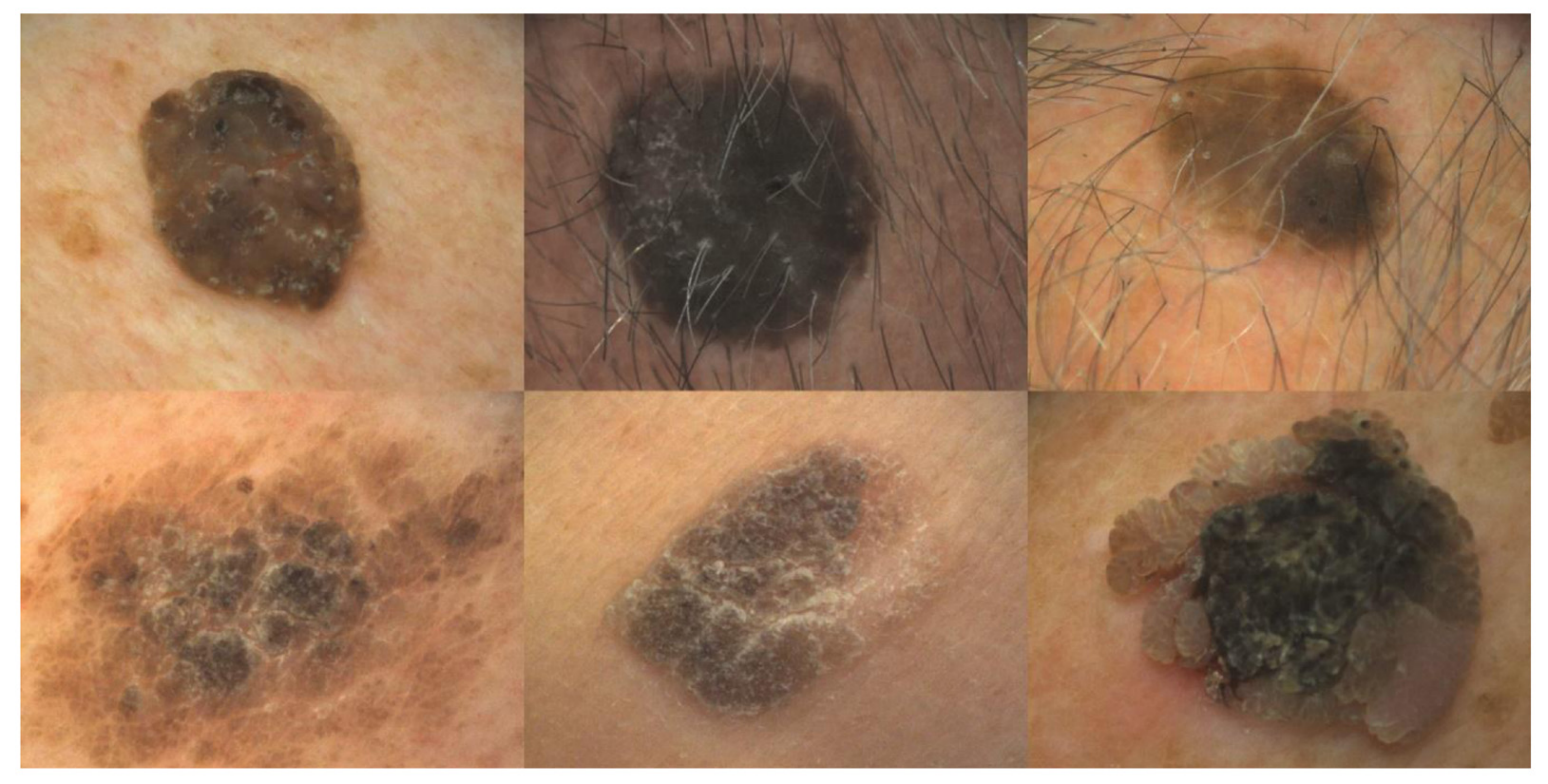
Hyperkeratotic seborrheic keratoses with horn cysts and pseudo-horn cysts (upper row) and without (lower row). This figure is an original image created by the Dr. K.T. Ashique for this publication and written informed consent was obtained from the patient.

A more sophisticated approach is the use of integrated digital dermoscopy analysis to gain objective measurements. The system evaluated 48 parameters to be studied as possible discriminant variables, grouped into four categories (geometries, colors, textures, and islands of color) integrated with three personal metadata items (sex, age, and site of lesion). Stepwise multivariate logistic regression of the data selected variables with the highest possible discriminant power ^9^. Although the system has been developed primarily for computer-aided melanoma diagnosis, SKs can also be identified. Examples using polarized light and a 16-fold magnification are shown in Figure 1 to Figure 4.

**Figure 2.**
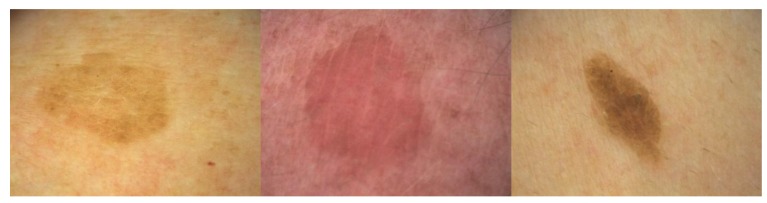
Reticulate seborrheic keratoses missing horn cysts. This type is more common in ultraviolet-exposed skin areas. This figure is an original image created by the author for this publication and written informed consent was obtained from the patient.

**Figure 3.**
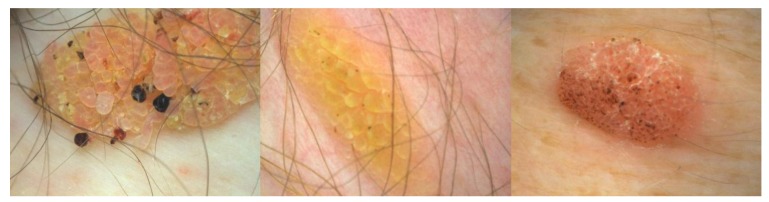
Clonal seborrheic keratoses. This figure is an original image created by the author for this publication and written informed consent was obtained from the patient.

**Figure 4.**
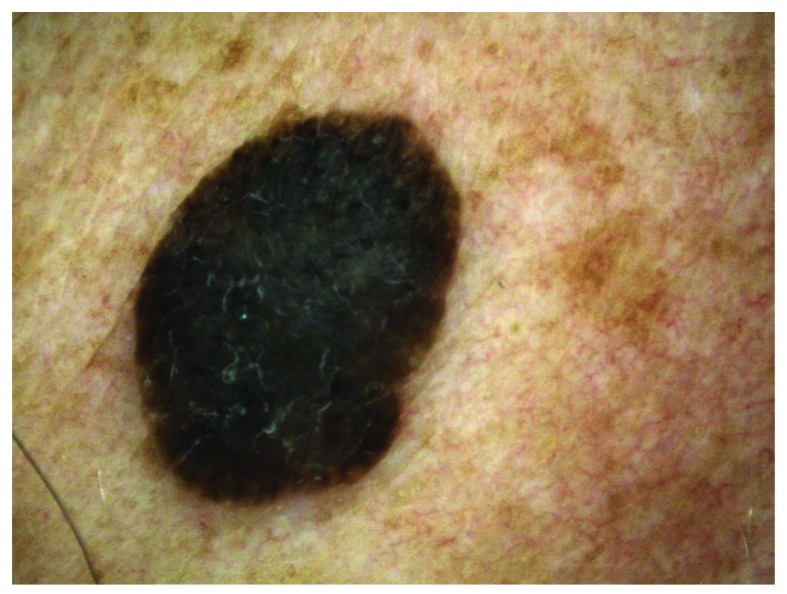
Melanoacanthoma. This figure is an original image created by the author for this publication and written informed consent was obtained from the patient.

Another non-invasive approach is autofluorescence analysis using an RGB (red, green, blue) smartphone camera and 405-nm light-emitting diode (LED) excitation. In a pilot trial, SKs could be identified and differentiated from basal cell carcinoma and melanocytic lesions ^10^.

Reflectance confocal microscopy (RCM) has been applied for non-invasive evaluation of skin tumors. In one study, 390 patients with a clinically suspicious diagnosis of SK were included for RCM ^11^. Dermatologists using this method could diagnose SK in 66.2% of patients but not in 24.9% of patients. The authors concluded that the great clinical variability of SKs and the restricted depth reached by RCM limit the use of this method for SK diagnosis ^11^.

## Differential diagnoses

Clinical differential diagnoses are (pigmented) basal cell carcinoma, pigmented Bowen’s disease, (verrucous) melanoma, extramammary Paget’s disease, common warts, acanthosis nigricans, and SK-like lesions localized to tattoo ^12– 14^. Although most SKs have a maximum diameter of less than 4 cm, sometimes giant lesions develop that raise some possible differential diagnoses, including Buschke–Löwenstein tumors ^3^.

## Histologic and clinical subtypes of seborrheic keratosis

SKs show a great clinical variability, but even on the histologic level, several subtypes can be defined: hyperkeratotic type, acanthotic type, reticular/adenoid type, clonal type, irritated type, melanoacanthoma, and verrucous SK with keratoacanthoma-like features ^1, 15^.

Dermatosis papulosa nigra is a clinical variant with multiple tiny lesions more common in patients with a Fitzpatrick skin type of at least 3 and in females. Some authors consider it a distinct entity; the histopathology, however, is identical to that of SK ^16^. Multiple eruptive SKs known as the Leser–Trélat sign have gained importance as a paraneoplastic disorder ^17, 18^.

## Pathogenesis

The etiopathology of SKs is not completely understood. They are considered a sign of aging skin in general and extrinsic aging, particularly due to chronic ultraviolet (UV) exposure. In contrast, a viral hypothesis suggesting the involvement of HPV has not been substantiated by recent studies ^19, 20^. Nevertheless, HPV p16 expression has been found in genital SKs at a frequency of between 65% and 69.6% ^21, 22^.

Amyloid precursor protein (APP) expression is higher in UV-exposed than in non-exposed skin sites and increases with age. The expression of APP has been evaluated in SKs versus normal skin by immunohistochemistry, Western blotting, and quantitative real-time polymerase chain reaction. APP and its downstream products (i.e. amyloid-β42) were more strongly expressed in SK than in paired adjacent normal skin tissues. In contrast, the expression of its key secretase (i.e. β-secretase 1) was low. The findings suggest that overexpression of APP may promote the onset of SK and is a marker of skin aging and UV damage ^23^.

Exome sequencing of SKs indicated three mutations per megabase pair of the targeted sequence. The mutational pattern depicted a typical UV signature, and the majority of alterations were C>T and CC>TT base changes at dipyrimidinic sites. Mutations of the tyrosine kinase fibroblast growth factor receptor 3 (FGFR3) were the most frequent; they were detected in 48% of lesions, followed by the PIK3CA (32%), TERT promoter (24%), and DPH3 promoter mutations (24%) ^24^. In other studies, SKs show mutations of FGFR3, such as R248C, S249C, G372C, S373C, A393E, K652E, and K652M, in 18% to 85% of lesions. Some of these mutations can be found in cervical cancer and urothelial bladder cancer, but in SKs they do not contribute to the proliferative activity of keratinocytes ^25^.

It could be demonstrated that SKs have frequently acquired oncogenic mutations in the receptor tyrosine kinase/phosphatidylinositol 3-kinase/Akt signaling cascade associated with a hypersensitivity to Akt inhibition. FoxN1 is a novel biomarker of the oncogenically activated but benign phenotype in SKs. **Neel *et al***. also established that Akt inhibition caused an increase in p53 protein expression, but not RNA expression, and that Akt-mediated apoptosis was dependent on p53 and FoxO3, a target of Akt ^26^. Merkel cell polyoma virus (MCPyV) has been detected by polymerase chain reaction and fluorescence *in situ* hybridization in six out of 23 SKs. p16 expression was not associated with the presence of MCPyV ^27^.

### Hyperkeratotic type

This is the most common subtype ( Figure 1). The tumors are characterized by huge acanthosis but mild or missing hyperkeratosis and papillomatosis. Horny invaginations that on cross-sections appear as “pseudo-horn cysts” are numerous. True horn cysts, which show complete keratinization with a very thin granular layer surrounding it, may also be seen ^1^.

### Reticular or adenoid type

The SKs of this subtype are characterized by reticular acanthosis of thin, double row with mild to moderate hyperkeratosis and papillomatosis. These SKs are often hyperpigmented. Horn pearls are uncommon. This subtype is more common in sun-exposed areas ( Figure 2) ^28^.

### Clonal type

In this type, marked acanthosis and papillomatosis are associated with orthohyperkeratosis. Tumor cells are spindle-shaped. With a demarcated tumor, islands of basaloid cell nests are found. Major differential diagnosis to clonal SK is pagetoid Bowen’s disease ( Figure 3). Immunohistochemistry may be helpful in selected cases to distinguish the two entities. Cytokeratin-10 is more highly expressed in clonal SK, whereas increased Ki-67–positive cells and the presence of more than 75% positive p16 cells are in favor of pagetoid Bowen’s disease ^29, 30^. Clonal nests of SKs can show a diffuse or patchy positivity for p16. A p16 positivity in clonal nests of SKs without concurrent atypical histologic features is typical for SK and not for pagetoid Bowen’s disease ^31^.

### Irritated type

**Figure 5.**
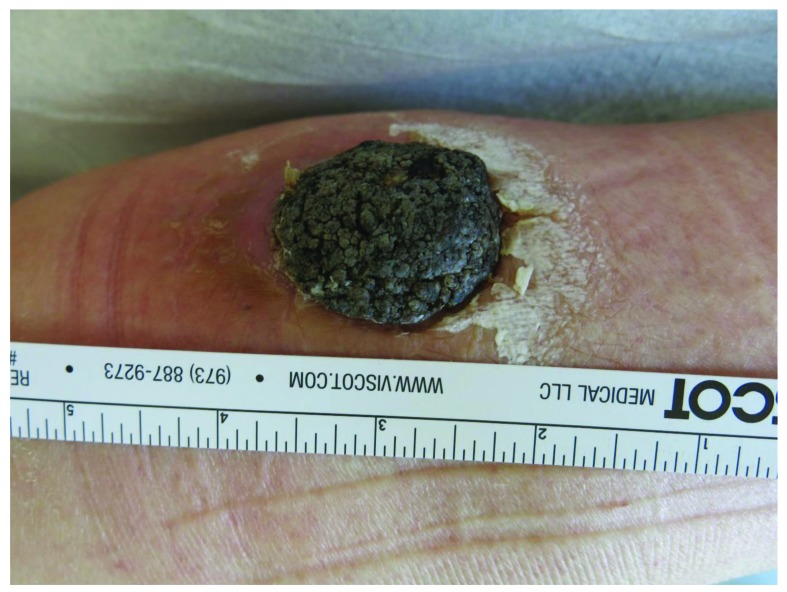
Irritated seborrheic keratosis resembling verrucous squamous cell carcinoma. This figure is an original image created by the author for this publication and written informed consent was obtained from the patient.

Here, a proliferation of spindle-shaped eosinophilic tumor cells is found, sometimes in whorl-like formations. Dyskeratotic cells may be present. In clinical practice, it is important to separate irritated SK from cutaneous squamous cell carcinoma (SCC) ( Figure 5). Immunohistochemistry may be helpful to distinguish the two entities. In a recent study, the combination of U3 small nucleolar ribonucleoprotein protein IMP3 and B-cell lymphoma 2 regulatory protein (Bcl-2) could be helpful in distinguishing between irritated SK and SCC in daily clinical practice whereas epidermal growth factor receptor (EGFR) immunohistochemistry did not appear to be useful in this setting ^32^.

### Melanoacanthoma

This is the hyperpigmented acanthotic type with a proliferation of basaloid cells with mild or even absent hyperkeratosis. Numerous melanocytes are intermingled. Melanophages are located within the dermis underneath a tumor ( Figure 4) ^1^. In contrast to common SKs, these lesions rarely occur on oral mucosa ^33^.

### Verrucous seborrheic keratosis with keratoacanthoma-like features

This subtype is rare. In one study, the frequency was estimated as 0.8% of all keratoacanthomas ^15^. In addition to showing the classic histologic features of SKs, these lesions show typical keratoacanthoma-like features such as a dome shape, marginal lips, a well-differentiated epidermis, and a crater with keratin plug formation. HPV16 was detected ^15^.

### Dermatosis papulosa nigra

**Figure 6.**
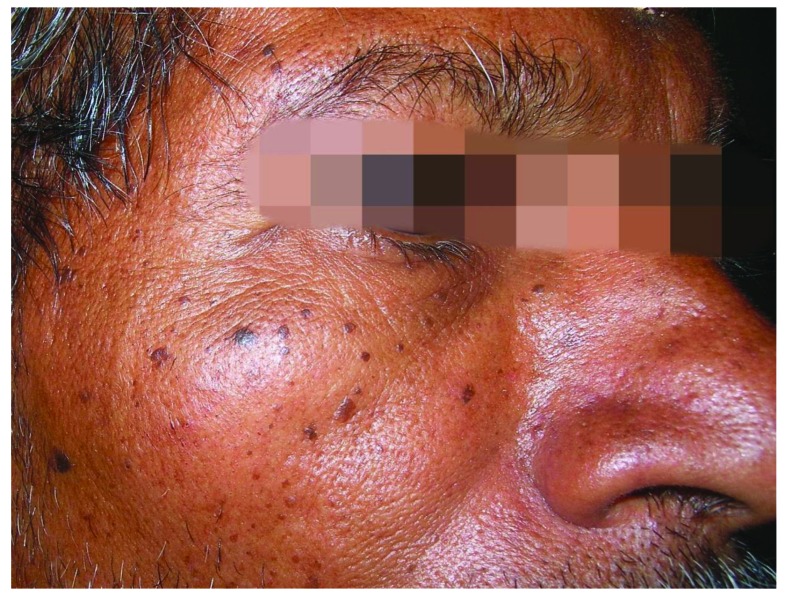
Dermatosis papulosis nigra. This figure is an original image created by the author for this publication and written informed consent was obtained from the patient.

In contrast to classic SKs, dermatosis papulosa nigra is more frequent in patients of African or Asian descent. It has been calculated that 10% of African-Americans are affected. A positive family history is seen in up to 85% of cases. Women are affected twice as often as males. The onset of dermatosis papulosa nigra is earlier than that of SKs. The papules are small and are most often found in UV-exposed areas ( Figure 6). The histology is similar to that of SKs of the acanthotic or reticular type. The lesions frequently harbor mutations of FGFR3, as do SKs ^16, 34, 35^.

### Leser–Trélat sign

The Leser–Trélat sign is characterized by the sudden appearance of multiple (often pruritic) SKs in association with an underlying malignancy. A great number of tumors, such as lung cancer, esophageal carcinoma, nasopharyngeal carcinoma, mycosis fungoides, Sézary syndrome, and plasmacytoma, have been described in association with this paraneoplastic disorder ^36– 41^. Tumor screening is recommended in case of abrupt development of multiple pruritic SKs.

### Pseudo-Leser–Trélat sign

The inflammation of pre-existing seborrheic warts during chemotherapy of malignancies using drugs such as cytarabine, docetaxel, gemcitabine, or PD1 inhibitors like nivolumab has been coined pseudo-Leser–Trélat sign ^42, 43^. It can be associated with burning and pruritus. Tumor therapy can be continued.

## Treatment

Patients have a wide range of motivations for treating or removing SKs, including embarrassment from the stigmatizing appearance of the lesion, physical irritation or pruritus, and a desire to look younger ^44^. Lesions that are inflamed, bleeding, ulcerated, or sufficiently irritated should be further characterized by a biopsy or excision to rule out malignancies ^45^.

In a study from the US, data were obtained from a survey of 594 practicing, board-certified dermatologists ^1^. On average, 155 patients with SKs are seen in practice, and one third exhibit more than 15 lesions. On average, these dermatologists treat 43% of SK cases with cryosurgery as the most common method. Other minor surgical options commonly employed are shave excision, electrodessication, curettage, or a combination of these ^1^. In another study, dermatologists managed 89% of SKs by using minor surgery compared with 51% by other specialties ^46^. Therefore, SK management by dermatologists is more cost-effective ^46^.

Patients preferred cryotherapy over curettage in a small trial (n = 25) ^47^, but physicians rating observed more redness at 6 weeks and the tendency for hypopigmented scar formation at over 12 months with curettage. Leftover SK lesion occurred more frequently with cryotherapy in the short and long term ^47^.

The use of laser surgery for the treatment of SKs has a long tradition. However, it is more expensive than other minor surgical methods. What is the additional benefit of using the more sophisticated tools?

A comparative trial was carried out in 42 patients with SKs of 0.5 to 3 cm located on the back, chest, face, and neck ^48^. Lesions with a similar size and location on the same patient were matched. In the same session, half of the lesions were treated with cryotherapy and the other half were treated with Er:YAG (erbium-doped yttrium aluminium garnet) laser ^48^.

Following the first treatment, complete healing was detected in all of the lesions (100%) treated with Er:YAG lasers whereas the healing rate was 68% in the cryotherapy group ( *P* <0.01). In the Er:YAG laser–treated group, hyperpigmentation was significantly lower than in the cryotherapy group ^48^. The results are in line with the author’s own experience with Er:YAG laser, where a lower rate of recurrence compared with shaving was noted ^49^, and a trial from the UK ^50^.

The CO _2_ laser is an effective alternative option but with a slightly higher risk of scarring and pigmentary changes ^51, 52^. Small case series have used intense pulsed light ^53^, pulsed-dye laser ^54^, Nd:YAG (neodymium-doped yttrium aluminum garnet) laser ^55^, or the 755-nm alexandrite picosecond laser ^56^ for SK removal. Larger trials, however, are missing. There is also a need for a head-to-head comparison of different laser types for SK removal to determine what the best choice is.

There is a need for improvement in SK treatment by surgical and medical approaches ^44^.

Two recent randomized trials (ClinicalTrials.gov identifiers: NCT02667236 and NCT02667275) compared the safety and efficacy of 40% hydrogen peroxide topical solution (HP40; Eskatat, Aclaris Therapeutics, Inc., Wayne, PA, USA) versus vehicle for the treatment of SKs. These trials included 937 patients with four SKs who were randomly assigned 1:1 to HP40 or vehicle ^57^. The clinical response was graded by using the Physician’s Lesion Assessment (PLA) scale (0: clear; 1: near-clear; 2: ≤1 mm thick; and 3: >1 mm thick). After one treatment, SKs with PLA greater than 0 were re-treated 3 weeks later. At day 106, significantly more HP40 patients versus vehicle achieved PLA of 0 on all four SKs (4% or 8% versus 0%) and three of four SKs (13% or 23% versus 0%). A higher mean per-patient percentage of SKs were clear (25% versus 2% or 34% versus 1%) and clear/near-clear (47% versus 10% or 54% versus 5%) with HP40 versus vehicle. Most local skin reactions, such as redness, burning, and stinging, were mild and resolved by day 106 ^57^.

The efficacy of this treatment was highest on facial lesions (clear or near-clear in 65%) followed by truncal SKs (46%) and SKs on the extremities (38%). Facial SKs also responded much quicker than lesions on other body parts probably because facial SKs are often thinner. Delayed adverse reactions such as pigmentary changes and scarring were least frequently reported for the facial SKs ^58^.

HP40 may act not only through its direct oxidation of organic tissues, generation of reactive oxygen species, and local lipid peroxidation but also by the generation of local concentrations of oxygen that are toxic to SK cells and induce apoptosis ^59, 60^. Compared with cryosurgery, HP40 seems to be less toxic to melanocytes, suggesting a better safety profile concerning induced pigmentary changes after the procedure ^61^.

An aqueous nitric–zinc complex solution has been developed for SK removal. The formulation contains nitric acid, zinc and copper salts, and organic acids (acetic, lactic, and oxalic acid). A clinical trial used the topical application of this nitric–zinc complex to obtain a whitening or yellowish reaction on top of SKs. Application of the nitric–zinc solution was performed every other week until clinical and dermoscopic clearance or crust formation for a maximum of four applications. All subjects, who reported no or minimal discomfort during and after the application of the solution, completed the study. After 8 weeks, complete clearance was observed in 37 of 50 lesions after an average of three applications per lesion. A partial response, with minimal persistent residual spots, was detected in the remaining 13 lesions. All patients with complete clearance showed no relapses at a 6-month follow-up ^62^.

Case reports with the successful use of diclofenac gel, imiquimod, dobesilate, or calcitriol have been published ^63– 66^. A meta-analysis of topical vitamin-D analogues concluded that these compounds are ineffective for SKs ^67^.

## Conclusion

SKs are the most common benign skin tumors in humans. The clinical variability of SKs can be confused with other potentially malignant skin diseases. Dermoscopy may aid correct diagnosis. Current understanding of their pathogenesis has not yet been translated into new treatment options. Minor surgeries are commonly used in dermatologic practice. Recently, clinical trials with topical HP40 suggested that this medical treatment could be an alternative, particularly for facial lesions ^68^.
